# SES inequalities in cause-specific adult mortality: a study of the long-term trends using longitudinal individual data for Sweden (1813–2014)

**DOI:** 10.1007/s10654-020-00685-6

**Published:** 2020-10-01

**Authors:** Enrico Debiasi, Martin Dribe

**Affiliations:** Centre for Economic Demography, Department of Economic History, Lund University, P. O. Box 7083, 220 07 Lund, Sweden

**Keywords:** Socioeconomic status, Cause-specific adult mortality, Sweden, Long-term trend

## Abstract

Higher socioeconomic status (SES) is associated with lower mortality, and this correlation has been confirmed using different indicators across several geographical settings. Nevertheless, the timing of the emergence of the SES gradient remains unclear. We used individual-level longitudinal data for a regional population in southern Sweden covering the period between 1813 and 2014, and we applied a cause-specific proportional hazard model. We estimated SES differences in all-cause, nonpreventable, preventable, and cause-specific adult mortality in four subperiods (1813–1921, 1922–1967, 1968–1989, 1990–2014) by gender adjusting for birth year, place of residence, marital status, and migration status. The SES gradient in mortality present today for both genders emerged only around 1970, and with few exceptions, it emerged at approximately the same time for all causes of death. It emerged earlier for women than for men, particularly in infectious diseases. In the nineteenth and early twentieth centuries, we found a positive association between SES and mortality from circulatory diseases for men. SES has not always been a fundamental cause of mortality; it only emerged as such in the second half of the twentieth century. We argue that habits and behaviors embedded in the different social strata played a major role in the emergence of the SES gradient.

**Electronic supplementary material:**

The online version of this article (10.1007/s10654-020-00685-6) contains supplementary material, which is available to authorized users.

## Background

Socioeconomic status (SES) is positively related to health and negatively related to mortality. In most developed societies today, there is a perfect mortality gradient by SES [1–3], including egalitarian societies with a developed welfare state, such as Sweden [4, 5]. Reducing socioeconomic inequalities is a high priority in public health [6], since it may improve life expectancy at the national level to a larger extent than eliminating cardiovascular diseases or cancer [7, 8]. The overwhelming and consistent evidence together with the substantial impact on public health make the study of this relationship of interest to policy makers [6, 9].

The mechanisms behind SES differentials in mortality are still debated. While several studies examined all-cause mortality [10, 11], focusing on specific causes of death can provide valuable insights in understanding these mechanisms [4, 12].

A much argued topic in the literature is when the SES gradient in mortality emerged [13–16]. While some studies have found SES differences in adult mortality in the distant past, others point towards a more recent emergence [17]. According to the Fundamental Cause Theory (FCT), inequalities in mortality by SES are more or less universal [18]. Even if the causal mechanisms vary historically and geographically, SES remains a key determinant of health and mortality [19, 20] even when taking demographic and epidemiological transitions [21] into account [22]. Several studies support this theory [23, 24], although most of these studies are based on data approximately from the 1970s onwards [24]. Findings from the late nineteenth and early twentieth centuries are more mixed [15, 17, 25–28]. The FCT emphasizes the importance of cause-specific mortality [22], but the evidence remains inconclusive [29–46].

Based on the FCT, we expect SES differences in adult mortality to have emerged from the end of the nineteenth century onwards and differences in cause-specific mortality to have emerged over time. Moreover, such changes should follow the preventability of causes of death, as higher SES groups are able to decrease their mortality risk through earlier access to prevention and interventions [18, 24, 47] because of higher education and income and better social context [48]. Several studies have found that SES is indeed more strongly related to preventable mortality [47–49]. Interestingly, SES differences have also been found also for nonpreventable causes [49, 50].

A study of SES mortality gradient emergence in Sweden is of particular interest because, since the last decades of the twentieth century, mortality inequalities by SES continue to be observed in one of the most equal countries in the world with an extensive welfare state; this is usually referred to as “the Nordic paradox” [51]. On the one hand, we could expect that the expansion of the welfare state during the twentieth century contributed to reduce mortality inequalities. On the other hand, previous studies have pointed towards a recent emergence of mortality inequalities regardless of the generous social policies [13, 17]. This pattern could suggest that the welfare state helped remove differentials related to material resources, but not those connected to, for example, psychosocial factors and lifestyle.

The aim of this study was to advance the understanding of mortality inequalities by studying SES differences in cause-specific adult mortality for men and women over a period of 200 years, focusing on when the SES gradient emerged, and in which causes of death. We estimated hazard ratios (HRs) for all-cause, nonpreventable, preventable, and cause-specific mortality in relation to SES separately by period and gender, adjusting for birth year, marital status, parish of residence, and migration status. To our knowledge, there is no published study analyzing the association between SES and cause-specific adult mortality over such a long period. This type of analysis has never been possible before and allowed us to shed light not only on the relationship between SES and cause-specific adult mortality but also on when and how it became the norm in the Western world. Taken together, our findings contribute to the knowledge about historical trends in mortality inequalities by extending the period of observation as far back as the early nineteenth century, and thus complementing literature that has shown conclusive evidence only for the period after the 1970s. A further key contribution lies in the analysis of specific causes of death, which provides a more detailed account of possible mechanisms at play and adds to a body of literature mainly focused on all-cause mortality.

## Methods

### Data

We used individual-level longitudinal data from the Scanian Economic-Demographic Database (SEDD) [52]. SEDD contains information for five rural and semi-urban parishes and a port town in the south of Sweden. Individuals have been followed across generations from 1813 until 2014. The data for the port town of Landskrona starts in 1922. Uniquely, the data encompass a period of 200 years for which cause-specific mortality can be studied longitudinally at the individual level.

For the period up to 1968, information about demographic events and occupation is derived from parish registers that were continuously updated with individual-level information for each household. For the period between 1968 and 2014, data are derived from administrative registers managed by Statistics Sweden (*SCB*) and the National Board of Health and Welfare (*Socialstyrelsen*), which have been linked to the historical data using personal identification numbers.

The analytical sample includes 180,744 individuals and 33,024 deaths. The sample size increased throughout time from 16,272 subjects in the first period (the Swedish population in the age groups of interest in 1875 was 1,819,054 [53]) to 67,284 subjects in the last period (5,221,046 people aged 30 to 89 in Sweden as of 1990 [53]). The population under study is not a statistically representative sample of Sweden. However, it does reflect similar economic and health conditions to most rural [54] and urban [55] areas at the time of the study [56]. Moreover, previous studies of total adult mortality found patterns of SES differences similar to Sweden as a whole [15, 17, 57]. Information on migration in and out of the area allowed for precise calculation of the population at risk. While the city of Landskrona experienced considerable net in-migration during much of the study period [55], the migration patterns in the rural area before 1970 were more circular. In-migrants and out-migrants were quite similar both in terms of SES and the places they came from/moved to [58]. Mass emigration from Sweden to North America took place between approximately 1860 and 1930 but was not particularly high from the area under study [59]. From 1930, Sweden became a country of net in-migration [53], and the area it mostly affected was the city of Landskrona, which added immigrants from Denmark, Finland, and Germany, among others [55]. We evaluate possible biases related to migrants being systematically different from non-migrants in terms of SES and mortality in a sensitivity analysis excluding all foreign born individuals.

The linkage of the historical (i.e. before 1968) and contemporary (i.e. since 1968) data allowed us to follow individuals in the area under study from 1968, even after migrating out to another place within Sweden, and their children and grandchildren throughout the country.

**Fig. 1:**
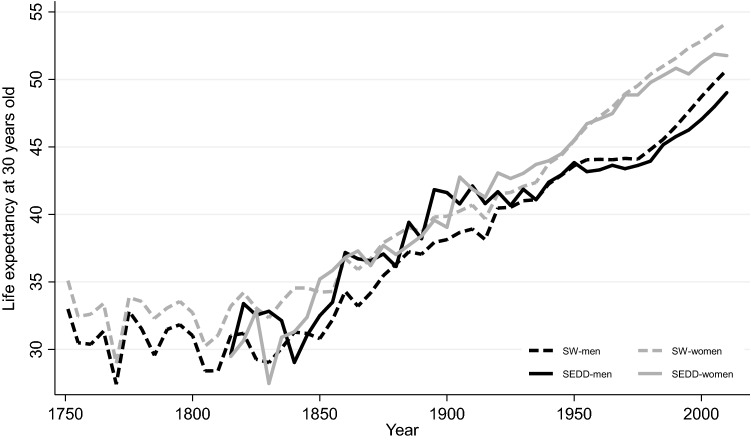
Period life expectancy at 30 years old in 5 years intervals for men (black) and women (gray). *Note*: Period life expectancy for Sweden (dashed) was calculated using data from the Human Mortality Database while for SEDD (solid) it was obtained by calculating the area under the Kaplan–Meier survival curve

We divided the data into four subperiods reflecting changes in data sources and availability, and in the societal and epidemiological context. The first period (1813–1921) encompassed a pre/early industrial society with mortality declining from pre-transitional levels to a continuously increasing life expectancy (Fig. 1). In the second period (1922–1967), the foundation of the Swedish welfare state was laid. The last two periods (1968–1989, 1990–2014) were characterized by continued economic growth and the consolidation and widening of welfare policies [60].

### Variables

Occupational status was updated annually between 1815 and 1968 and between 2001 and 2011 (the occupation in the last 3 years is assumed to be the same as in 2011); in the period in between, occupation is available at census years (1970, 1975, 1980, 1985, 1990). Occupational notations have been coded in HISCO [61] and grouped into a 12-category classification: HISCLASS [62]. In the analysis, we aggregated HISCLASS categories into three groups: nonmanual, manual, and farmers. We also ran the analysis on a six-category grouping. While it may be problematic to use the same SES classification over 200 years, it has been shown that occupational hierarchies remain quite stable over time, particularly when using broad SES groups [63].

SES was used as a time-varying variable until the age of 65. Between 65 and 90 years of age, we considered the highest SES between ages 50 and 65, because it should represent the SES at the peak. Moreover, SES after age 65 could be misleading because of retirement. Given this approach to determine SES in old age, only individuals observed before age 65 were included in the analytical sample. For married individuals, we selected the highest SES within the couple. All individuals were under observation until death, out-migration (outside the parishes before 1968 and outside Sweden after 1968), or the end of 2014.

The outcome variable was cause-specific adult mortality (ages 30 to 90). For the period before 1968, the causes of death that were originally recorded as text strings in the parish registers, have been translated into ICD-10 codes [64]. The causes of death for the period after 1968 contained in the administrative register (*Dödsorsaksregistret)* were coded in ICD-8 between 1969 and 1986, in ICD-9 between 1987 and 1996, and in ICD-10 from 1997 onwards.

We grouped ICD codes according to two classifications. First, we divided causes of death into preventable and nonpreventable following the Avoidable Mortality in the European Union (AMIEHS) classification (see also [50]). Second, since the preventability of diseases has changed over time, we added a more stable and objective measure of cause-specific mortality, following ICD chapters: (1) infectious and parasitic diseases (including pneumonia and influenza); (2) circulatory diseases; (3) respiratory diseases (including lung, larynx, trachea, bronchus, lip, oral cavity, and pharynx cancers); (4) other cancers; (5) external causes; (6) other and ill-defined causes of death; and (7) missing causes of death.

### Analysis

To estimate differences in cause-specific mortality by SES and how they have developed over time, we used a cause-specific proportional hazard model, which estimated the effect of covariates on the mortality risk from a specific cause. Each model represented a separate Cox regression in which the event corresponded to a specific cause of death. Individuals who died from a different cause were right censored.

The exponential of the regression coefficient represents the cause-specific mortality risk in the group under consideration compared to the reference category [65]. The cause-specific proportional hazards model is well suited for etiological studies [66–68].

The analysis was performed separately by period and gender. We further adjusted for birth year, marital status (never married, married, and previously married), parish of residence (grouped by geographical proximity) and migration status in the second, third, and fourth periods. In the initial period, there were very few people born outside Sweden. We evaluated the assumption of proportional hazards using a test based on the scaled Schoenfeldt residuals (‘estat phtest’ in STATA). For preventable diseases in the third and last periods, the test indicated non-proportionality for both men and women, mainly affecting the NA category. A log–log plot indicated that the hazard lines for nonmanual and manual occupations were parallel, except for in very early ages. The same held true for nonpreventable mortality in the last two periods for women. We therefore ran a sensitivity analysis by dividing the sample into two age groups. We further verified the robustness of our results with respect to the exposure (e.g., using individual SES regardless of marital status) and outcome (e.g., using a different classification of preventability).

## Results

Table 1 shows the descriptive statistics of the analyzed sample by gender. The changing SES structure over time, with an increasing share of nonmanual workers and a decreasing share of farmers, indicated the large societal changes taking place during the period under consideration, as Sweden shifted from an agricultural society into a developed welfare society [60].

**Table 1:** Descriptive statistics for men and women, 30 to 90 years old (five parishes and, from 1922, Landskrona)

	Men				Women			
	1813–1921	1922–1967	1968–1989	1990–2014	1813–1921	1922–1967	1968–1989	1990–2014
SES-6 (%)								
Higher managers/professionals	2.5	8.1	7.8	10.8	2.5	7.6	7.6	9.4
Lower managers/professionals/clerical	6.4	20.4	38.3	39	5.5	23.4	39.8	42
Foremen and medium skilled workers	10	26.7	20.5	15.2	9.2	23.1	17.1	10
Lower skilled workers/farm workers	25.9	24.1	24	22.8	34.5	26.3	23.3	24.2
Unskilled workers/farm workers	27.5	15.5	3.8	3.8	22.9	12.2	6.4	7.3
Farmers and fishermen	24.2	4.4	3	1.4	20.4	3.8	2.6	1.2
NA	3.6	0.8	2.6	7	5.1	3.6	3.2	6
SES-3 (%)								
Nonmanual	8.8	28.5	46.1	49.9	7.9	31	47.4	51.4
Manual	63.4	66.3	48.3	41.8	66.6	61.6	46.8	41.4
Farmers	24.2	4.4	3	1.4	20.4	3.8	2.6	1.2
NA	3.6	0.8	2.6	7	5.1	3.6	3.2	6
Civil status (%)								
Never married	16.2	18.9	13.7	22.6	18	23.3	9.1	14.8
Currently married	73.4	75.1	73.1	58	65.4	66	63.9	52.5
Previously married	10.4	6	13.3	19.4	16.6	10.7	27	32.7
Migrant status (%)								
Born in Sweden	98.8	95.6	87.4	81.3	99.1	95.9	88.7	81.4
Born outside Sweden	1.2	4.4	12.6	18.7	0.9	4.1	11.3	18.6
Parish of residence (%)								
Hög, Kävlinge	28.6	12.4	11.2	14.6	28.1	12.1	11.1	15
Halmstad, Sireköpinge, Kågeröd	71.4	11.5	6.6	6.3	71.9	10.1	5.8	5.7
Landskrona	0	76.1	82.2	79.1	0	77.8	83.1	79.3
Cohort (mean)								
Birth year	1827.3	1897.6	1925.8	1948.1	1827.3	1896.5	1923.8	1945.9
Cause of death group (%)								
Infectious and parasitic	14.5	10.2	3.4	3.8	14	10.4	4.4	4.5
Circulatory system	4.9	38.3	52.4	42.7	4.7	39.1	49.9	41.2
Respiratory system and lung cancer	6.8	4.3	8.1	11.8	5.3	3.1	4.9	9.5
Other cancers	2.4	17.1	19	21.8	4.2	19.9	24.9	23.8
External causes	4.1	8.4	7.6	5.2	0.8	3	4.5	3.4
Other and ill-defined causes	35.7	20.7	9.1	14.7	36.8	23.6	11.4	17.5
Missing	31.6	0.9	0.2	0.1	34.2	0.8	0.1	0.1
Cause of death preventable (%)								
Nonpreventable causes	23	28.8	25.6	32.9	20.3	27	26.3	34.9
Preventable causes	18	61.6	73.9	65.8	16.6	60.6	73.5	64.5
Ill-defined causes	27.4	8.6	0.3	1.2	28.9	11.6	0.1	0.6
Missing	31.6	0.9	0.2	0.1	34.2	0.8	0.1	0.1
Deaths	2009	4633	4786	6115	2129	4330	3699	5323
Time at risk	99,016.7	344,954.4	274,102.4	379,639	104,349.9	366,401.3	291,272.2	399,771.6

The cause-of-death groups partially reflected the shift in the leading causes from infectious diseases to man-made diseases, particularly when looking at groups (1), (2), and (3).

Tables 2 and 3 report hazard ratios for all-cause mortality and nonpreventable and preventable mortality, respectively, for men and women separately. The results for all-cause mortality in the last two periods showed a clear SES gradient. Interestingly, in the second period, male all-cause mortality was positively associated with SES: the nonmanual occupations group had a 15% higher mortality risk (HR 1.145, 95% CI 1.071–1.223) than the manual workers. While this held for men, mortality in women was lower in nonmanual occupations beginning in the second period, and the SES differences increased in the most recent period.

**Table 2:** All-cause and nonpreventable versus preventable mortality hazard ratios and 95% confidence intervals, men, ages 30–90 (five parishes and, from 1922, Landskrona)

	All-cause mortality				Nonpreventable causes				Preventable causes			
	1813–1921	1922–1967	1968–1989	1990–2014	1813–1921	1922–1967	1968–1989	1990–2014	1813–1921	1922–1967	1968–1989	1990–2014
Nonmanual	0.883	1.145	0.696	0.571	1.117	1.106	0.712	0.604	1.299	1.150	0.694	0.556
	[0.732, 1.065]	[1.071, 1.223]	[0.653, 0.742]	[0.540, 0.604]	[0.771, 1.617]	[0.978, 1.251]	[0.627, 0.809]	[0.548, 0.665]	[0.927, 1.821]	[1.057, 1.250]	[0.645, 0.747]	[0.519, 0.596]
Manual (ref.)												
Farmer	0.884	1.149	0.747	0.694	1.099	0.99	0.861	0.656	0.827	1.209	0.714	0.719
	[0.795, 0.983]	[0.997, 1.325]	[0.638, 0.875]	[0.601, 0.803]	[0.889, 1.358]	[0.749, 1.309]	[0.635, 1.167]	[0.501, 0.858]	[0.633, 1.081]	[1.003, 1.457]	[0.593, 0.860]	[0.605, 0.855]
NA	1.107	1.076	0.862	1.073	1.324	1.361	1.176	1.309	1.427	0.920	0.718	0.954
	[0.871, 1.406]	[0.822, 1.408]	[0.658, 1.130]	[0.926, 1.242]	[0.852, 2.058]	[0.860, 2.154]	[0.766, 1.807]	[1.034, 1.657]	[0.862, 2.363]	[0.628, 1.349]	[0.503, 1.024]	[0.787, 1.156]
Subjects	8141	25,354	24,369	34,266	8141	25,354	24,369	34,266	8141	25,354	24,369	34,266
Failures	2009	4633	4786	6115	463	1336	1227	2011	361	2856	3536	4024
Time at risk	99,016.7	344,954.4	274,102.4	379,639	99,016.7	344,954.4	274,102.4	379,639	99,016.7	344,954.4	274,102.4	379,639

Time at risk in person years

**Table 3:** All-cause and nonpreventable versus preventable mortality hazard ratios and 95% confidence intervals, women, ages 30–90 (five parishes and, from 1922, Landskrona)

	All-cause mortality				Nonpreventable causes				Preventable causes			
	1813–1921	1922–1967	1968–1989	1990–2014	1813–1921	1922–1967	1968–1989	1990–2014	1813–1921	1922–1967	1968–1989	1990–2014
Nonmanual	0.848	0.906	0.675	0.635	0.919	0.944	0.682	0.667	0.853	0.901	0.672	0.618
	[0.694, 1.036]	[0.843, 0.974]	[0.626, 0.727]	[0.598, 0.675]	[0.586, 1.443]	[0.824, 1.080]	[0.590, 0.788]	[0.603, 0.739]	[0.561, 1.297]	[0.821, 0.988]	[0.616, 0.733]	[0.572, 0.666]
Manual (ref.)												
Farmer	0.942	0.993	0.629	0.589	1.298	0.847	0.731	0.523	0.988	1.075	0.59	0.618
	[0.840, 1.057]	[0.854, 1.155]	[0.500, 0.791]	[0.480, 0.723]	[1.034, 1.630]	[0.621, 1.155]	[0.485, 1.102]	[0.362, 0.755]	[0.750, 1.300]	[0.881, 1.311]	[0.448, 0.779]	[0.482, 0.793]
NA	1.225	1.082	0.858	1.150	1.209	1.199	0.723	1.314	1.268	1.077	0.905	1.065
	[0.999, 1.501]	[0.957, 1.224]	[0.708, 1.040]	[1.005, 1.316]	[0.785, 1.861]	[0.943, 1.525]	[0.485, 1.078]	[1.053, 1.641]	[0.784, 2.050]	[0.919, 1.262]	[0.726, 1.128]	[0.897, 1.263]
Subjects	8131	24,213	23,252	33,018	8131	24,213	23,252	33,018	8131	24,213	23,252	33,018
Failures	2129	4330	3699	5323	432	1170	971	1856	353	2624	2720	3431
Time at risk	104,350	366,401	291,272	399,772	104,350	366,401	291,272	399,772	104,350	366,401	291,272	399,772

Time at risk in person years

Results for nonpreventable and preventable mortality indicated that, for both genders, nonmanual workers and farmers had an advantage in the last two periods, regardless of preventability. These findings are consistent with the pattern found for the entire country [50]. In the second period there was a positive association between SES and preventable mortality for men. While women in the nonmanual group had lower mortality from preventable causes (HR 0.901, 95% CI 0.821–0.988), men in this group had higher mortality (HR 1.150, 95% CI 1.057–1.250). Supplementary Tables 1 and 2 (supplementary material) report the estimates for the ill-defined causes and for the missing group.

In Tables 4 and 5 we present the estimates for cause-specific mortality using the more detailed subdivision. For infectious and parasitic diseases, we found evidence of lower mortality for nonmanual workers for the last two periods (HR 0.686, 95% CI 0.480–0.979 and HR 0.531, 95% CI 0.394–0.716 for the third and fourth periods, respectively). A similar pattern was found in studies analyzing data covering the whole country [4, 46]. For men in the earlier periods, the coefficients suggested a lower mortality for nonmanual workers, but this difference was smaller in magnitude with larger confidence intervals (HR 0.839, 95% CI 0.515–1.368 and HR 0.948, 95% CI 0.759–1.183 for the first and second periods respectively). For women, while we did not find any association in the first period, nonmanual workers in the second period showed a lower mortality risk (HR 0.737, 95% CI 0.581–0.936).

**Table 4:** Cause-specific mortality hazard ratios and 95% confidence intervals, men, ages 30–90 (five parishes and, from 1922, Landskrona)

	Infectious and parasitic				Circulatory system				Respiratory system and lung cancer			
	1813–1921	1922–1967	1968–1989	1990–2014	1813–1921	1922–1967	1968–1989	1990–2014	1813–1921	1922–1967	1968–1989	1990–2014
Nonmanual	0.839	0.948	0.686	0.531	2.564	1.248	0.748	0.576	0.883	1.094	0.535	0.477
	[0.515, 1.368]	[0.759, 1.183]	[0.480, 0.979]	[0.394, 0.716]	[1.549, 4.242]	[1.124, 1.385]	[0.686, 0.816]	[0.529, 0.627]	[0.414, 1.957]	[0.799, 1.497]	[0.426, 0.672]	[0.404, 0.563]
Manual (ref.)												
Farmer	1.026	1.214	0.738	0.946	0.631	1.032	0.750	0.689	0.792	1.174	0.584	0.342
	[0.781, 1.348]	[0.781, 1.888]	[0.341, 1.601]	[0.524, 1.708]	[0.351, 1.136]	[0.809, 1.316]	[0.604, 0.930]	[0.557, 0.851]	[0.531, 1.180]	[0.584, 2.360]	[0.313, 1.089]	[0.187, 0.627]
NA	1.142	1.759	0.881	1.331	1.101	0.766	0.796	0.843	1.071	0.670	0.929	1.141
	[0.630, 2.070]	[1.001, 3.093]	[0.212, 3.668]	[0.671, 2.640]	[0.393, 3.089]	[0.442, 1.327]	[0.513, 1.233]	[0.646, 1.099]	[0.464, 2.468]	[0.0928, 4.837]	[0.374, 2.306]	[0.777, 1.675]
Subjects	8141	25,354	24,369	34,266	8141	25,354	24,369	34,266	8141	25,354	24,369	34,266
Failures	291	474	163	233	99	1775	2510	2609	136	199	390	719
Time at risk	99,016.7	344,954.4	274,102.4	379,639	99,016.7	344,954.4	274,102.4	379,639	99,016.7	344,954.4	274,102.4	379,639

	Other cancers			External causes				Other and ill-defined causes			
	1922–1967	1968–1989	1990–2014	1813–1921	1922–1967	1968–1989	1990–2014	1813–1921	1922–1967	1968–1989	1990–2014
Nonmanual	1.203	0.794	0.642	0.672	0.744	0.434	0.499	0.787	1.200	0.652	0.565
	[1.028, 1.407]	[0.689, 0.916]	[0.571, 0.722]	[0.265, 1.705]	[0.582, 0.951]	[0.335, 0.562]	[0.384, 0.647]	[0.556, 1.112]	[1.033, 1.394]	[0.523, 0.812]	[0.487, 0.655]
Manual (ref.)											
Farmer	1.099	0.837	0.819	1.147	0.981	0.736	1.248	0.894	1.385	0.704	0.625
	[0.790, 1.530]	[0.593, 1.181]	[0.605, 1.108]	[0.684, 1.924]	[0.508, 1.895]	[0.385, 1.409]	[0.666, 2.337]	[0.751, 1.063]	[1.054, 1.820]	[0.401, 1.237]	[0.415, 0.941]
NA	0.683	0.693	1.254	2.040	0.433	0.873	1.215	1.203	1.544	1.109	1.112
	[0.282, 1.655]	[0.340, 1.411]	[0.897, 1.755]	[0.851, 4.889]	[0.107, 1.746]	[0.467, 1.629]	[0.763, 1.933]	[0.811, 1.783]	[0.983, 2.425]	[0.538, 2.287]	[0.793, 1.560]
Subjects	25,354	24,369	34,266	8141	25,354	24,369	34,266	8141	25,354	24,369	34,266
Failures	792	909	1333	82	388	366	317	718	961	437	896
Time at risk	344,954.4	274,102.4	379,639	99,016.7	344,954.4	274,102.4	379,639	99,016.7	344,954.4	274,102.4	379,639

Time at risk in person years. In the first period, estimates for mortality from other cancers were not calculated as there was only a small number of cases

**Table 5:** Cause-specific mortality hazard ratios and 95% confidence intervals, women, ages 30–90 (five parishes and, from 1922, Landskrona)

	Infectious and parasitic				Circulatory system				Respiratory system and lung cancer			
	1813–1921	1922–1967	1968–1989	1990–2014	1813–1921	1922–1967	1968–1989	1990–2014	1813–1921	1922–1967	1968–1989	1990–2014
Nonmanual	1.082	0.737	0.620	0.741	1.368	0.945	0.670	0.668	0.150	0.832	0.635	0.551
	[0.662, 1.771]	[0.581, 0.936]	[0.426, 0.902]	[0.559, 0.982]	[0.729, 2.567]	[0.843, 1.061]	[0.602, 0.746]	[0.607, 0.735]	[0.020, 1.085]	[0.556, 1.245]	[0.456, 0.885]	[0.453, 0.669]
Manual (ref.)												
Farmer	1.179	1.110	0.570	0.266	1.043	1.258	0.806	0.661	1.026	0.485	0.161	0.547
	[0.888, 1.564]	[0.741, 1.662]	[0.222, 1.465]	[0.065, 1.082]	[0.611, 1.779]	[0.999, 1.584]	[0.598, 1.087]	[0.492, 0.889]	[0.652, 1.615]	[0.145, 1.622]	[0.022, 1.171]	[0.256, 1.169]
NA	1.261	0.841	0.716	1.769	1.824	1.053	0.897	1.271	0.902	1.241	0.473	0.577
	[0.738, 2.153]	[0.558, 1.267]	[0.261, 1.962]	[0.983, 3.181]	[0.793, 4.193]	[0.864, 1.282]	[0.684, 1.176]	[1.035, 1.561]	[0.397, 2.047]	[0.629, 2.449]	[0.171, 1.309]	[0.330, 1.010]
Subjects	8131	24,213	23,252	33,018	8131	24,213	23,252	33,018	8131	24,213	23,252	33,018
Failures	297	451	161	241	101	1692	1844	2193	112	136	180	508
Time at risk	104,349.9	366,401.3	291,272.2	399,771.6	104,349.9	366,401.3	291,272.2	399,771.6	104,349.9	366,401.3	291,272.2	399,771.6

	Other cancers				External causes			Other and ill-defined causes			
	1813–1921	1922–1967	1968–1989	1990–2014	1922–1967	1968–1989	1990–2014	1813–1921	1922–1967	1968–1989	1990–2014
Nonmanual	0.885	0.950	0.692	0.647	0.884	0.680	0.515	0.850	0.886	0.687	0.598
	[0.426, 1.904]	[0.814, 1.110]	[0.599, 0.799]	[0.572, 0.730]	[0.591, 1.323]	[0.482, 0.960]	[0.367, 0.723]	[0.607, 1.191]	[0.760, 1.033]	[0.552, 0.855]	[0.516, 0.694]
Manual (ref.)											
Farmer	0.805	0.713	0.413	0.606	0.431	0.612	0.882	0.870	0.918	0.622	0.473
	[0.436, 1.485]	[0.464, 1.096]	[0.240, 0.711]	[0.398, 0.925]	[0.100, 1.859]	[0.188, 1.993]	[0.318, 2.443]	[0.716, 1.056]	[0.685, 1.229]	[0.312, 1.241]	[0.277, 0.809]
NA	1.851	1.282	0.776	1.052	1.806	1.352	0.604	1.057	1.050	0.829	1.345
	[0.843, 4.066]	[0.969, 1.695]	[0.516, 1.169]	[0.786, 1.408]	[0.940, 3.469]	[0.684, 2.674]	[0.285, 1.282]	[0.739, 1.512]	[0.821, 1.344]	[0.471, 1.461]	[1.003, 1.803]
Subjects	8131	24,213	23,252	33,018	24,213	23,252	33,018	8131	24,213	23,252	33,018
Failures	90	862	920	1267	132	168	181	784	1024	423	930
Time at risk	104,349.9	366,401.3	291,272.2	399,771.6	366,401.3	291,272.2	399,771.6	104,349.9	366,401.3	291,272.2	399,771.6

Time at risk in person years. In the first period, estimates for mortality from external causes were not calculated as there was only a small number of cases

Among men, in the last two periods there were clear differences in mortality from circulatory diseases by SES, which widened from the 1968–1989 period (HR 0.748, 95% CI 0.686–0.816) to the final period (HR 0.576, 95% CI 0.529–0.627). Other studies of the entire country for the same period showed a similar overall pattern [12, 46, 69]. Interestingly, in the first two periods, nonmanual workers had a higher mortality (HR 1.248, 95% CI 1.124–1.385 for the second period and HR 2.564, 95% CI 1.549–4.242 for the first period). Women showed a similar outcome for the most recent period. The difference with respect to men was that, beginning in the 1920s, women in the nonmanual group had a lower risk of dying than women in the manual category.

For respiratory diseases, the period after 1968 was again characterized by lower mortality in the nonmanual category for both men and women. Before 1968, results showed small differences by SES. The only exception was for mortality due to other cancers for men in the first part of the twentieth century (HR 1.203, 95% CI 1.028–1.407). In this instance the most frequent diseases were malignant neoplasms of the stomach, prostate, rectum, colon, and pancreas (approximately 60% of cases in total).

When looking at external causes, in the last two periods, the difference between the nonmanual and manual categories was particularly evident, but clear inequalities were present throughout the period of analysis. Similar results were found by Kunst et al. [69] and Toch-Marquardt et al. [12]. The SES advantage in mortality from external causes was present in the earlier period as well (for women in the first period, the number of events was too small for a meaningful analysis).

### Analysis with the six-category SES classification and sensitivity analyses

**Fig. 2:**
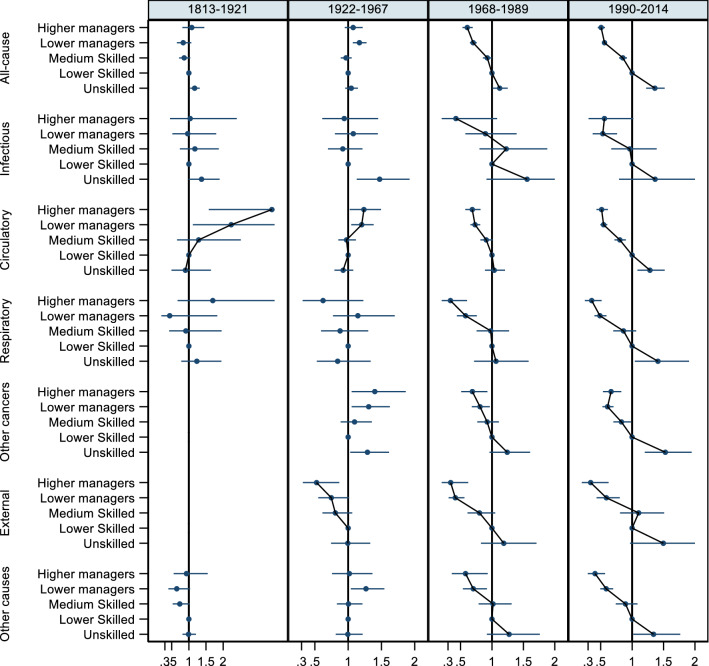
All-cause and cause-specific mortality hazard ratios and 95% confidence intervals using a more detailed SES grouping, men, ages 30–0 (five parishes and, from 1922, Landskrona). The reference category is lower skilled (estimates for farmers not shown)

**Fig. 3:**
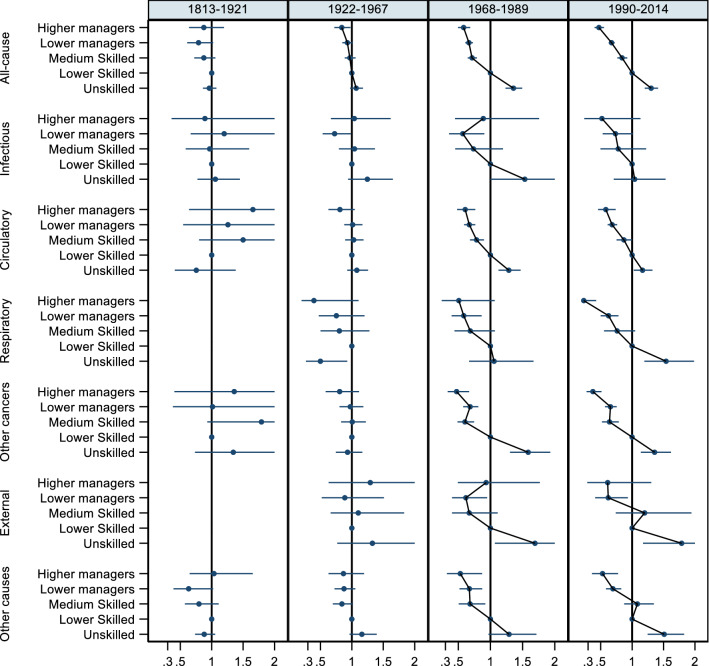
All-cause and cause-specific mortality hazard ratios and 95% confidence intervals using a more detailed SES grouping, women, ages 30–90 (five parishes and, from 1922, Landskrona). The reference category is lower skilled (estimates for farmers not shown)

We performed further analyses of preventable versus nonpreventable mortality and cause-specific mortality using a more detailed SES grouping (Supplementary Tables 3–6). The results for cause-specific mortality using the more detailed SES categorization are also shown in Figs. 2 and 3 for men and women, respectively. In these Figures, we highlight that the SES gradient is evident only in the later periods (estimates for farmers are not reported in the graphs in order to show more clearly the gradient from white to blue collar groups).

To verify the robustness of our findings, we ran a set of sensitivity analyses that took into account variations in the sample (Tables A1–A4), possible biases introduced by international migration (Tables A5–A6) and the definition of the exposure from family SES to individual SES (Tables A7–A8). We also ran the models dividing the sample into age groups in order to account for possible variations in mortality differentials by age (Tables A9–A12). Furthermore, we applied an alternative classification of preventability, namely, the one provided in Phelan et al. [47] (Tables A13–A14), and a different SES measure using HISCAM scores as a continuous variable [70] as well as HISCAM quartiles calculated by sex and periods (Tables A15–A18). All the estimates are shown in the supplementary material. The results and patterns described above were robust and did not fundamentally change in any of the tests.

## Discussion

This is the first study to analyze SES inequalities in cause-specific adult mortality for a period of 200 years using a comparable SES classification and longitudinal individual-level data. In accordance with previous studies, our findings demonstrated a consistent pattern of mortality advantage for higher-SES groups for both men and women from approximately 1970 onwards. Higher-SES groups showed a lower mortality risk both for nonpreventable and preventable mortality, and similar results have been found in previous studies [50]. While the lack of variance between nonpreventable and preventable causes could indicate that the grouping of causes of death was not well-suited to capture factors affecting SES groups differently, the pattern did not change when using another grouping following Phelan et al. [47].

In terms of the development of SES inequalities in cause-specific mortality over time, we found that the current SES gradient has not been present for very long. Only in the last 50 years did higher-SES groups have a clear advantage compared to lower-SES groups for both genders. Furthermore, the emergence of the SES gradient is a recent phenomenon that happened roughly at the same time for all causes of death considered.

When examining causes of death, we found a particularly interesting pattern for circulatory diseases in which higher-SES men showed higher mortality in the first period that decreased in the second period and developed into an advantageous position in the last two periods. A possible explanation for such a mortality trend, which was also present for other cancers and other causes of death, is related to behavior and lifestyle differences between SES groups that changed dramatically throughout the period. An unhealthy diet, smoking, alcohol consumption, and a sedentary lifestyle are often described as potential causes of the mortality gradient, as unhealthy habits are more common in lower-SES groups [2, 3]. The same logic can be applied to historical contexts, although the difference is that unhealthy behavior was more common in the higher-SES groups [15]. Similarly, Razzell and Spence [71] focused on behavioral characteristics to explain health differences by SES in pre-twentieth century England. Moreover, men were more exposed to these risks than women [16], which could explain the gender differences that we found in mortality from circulatory diseases. Especially in the first two periods, early-life conditions could also have played a role in explaining these results. To the extent that lower SES was associated with higher mortality in early ages, it may have selected stronger individuals who therefore had lower mortality as adults. However, a previous study using sibling fixed-effects models to control for the early-life environment found the same reverse association between SES and life expectancy at age 40 (and age 60), which does not suggest that selection mechanisms can explain this pattern [15].

We also found that the SES differences emerged earlier for women than for men, in particular in infectious diseases. In the second period (1922–1967), nonmanual workers already showed a lower mortality risk while we found no differences for men. One explanation for this pattern could be the fact that working class women had less bargaining power within the household, leading them to be discriminated against in terms of nutrition, and hence being more severely affected by infectious diseases [72, 73]. The second period also saw an important reduction in maternal mortality due to improvements in hygienic conditions, institutionalized maternal health care, and the introduction of sulfa drugs [74]. If these improvements benefited women with higher SES more than it did women with low SES, it could explain the earlier emergence of SES differences, particularly for infectious diseases. However, previous studies of the nineteenth century have found no class differences in maternal mortality [75], and when preventive services for maternal health spread on a large-scale throughout the country, it seemed to have especially benefited lower-SES groups [76].

One might be concerned that the dramatic changes in the SES structure over such a long period of time could affect the interpretation of the time trends. However, as we measured relative mortality differentials, the size of different SES groups has no impact on the measured differentials. The sensitivity analysis using quintiles of a continuous SES measure (HISCAM) also showed the same trends over time as the class scheme. Moreover, the emerging mortality gradient implies that is was not only a small group of low-status people that had high mortality, but that there were pronounced differentials over the entire SES structure.

As mentioned above and shown in the supplementary materials, our results are robust throughout a series of sensitivity analyses. However, a number of limitations should be considered. A common limitation in analyses of cause-specific mortality stems from the reliability of the causes of death. For the period after 1968, for which data is taken from national administrative registers, this may be a problem, particularly when the death is due to more than one disease. However, it has been shown that the quality of information contained in the Swedish cause of death register is relatively high [77] and that when causes of death are grouped at the chapter level (as was mainly done in this paper), the accuracy is even higher [78]. For the period before 1968 for which data is based on historical population registers, we have reasons to believe that the reporting of causes of death was fairly reliable, since the person in charge of reporting the cause of death was either a doctor or, particularly in the first period, the priest who received basic medical training, and in rural areas where a medical doctor was not present, they were the point of reference for health issues. Before the early twentieth century, when the reporting of the underlying causes of death was requested for every deceased individual, there was a higher proportion of people with missing information. In our study this affected only the first period, and in terms of differences by SES, only men (column 3 in Supplementary Table 1).

## Conclusions

SES has not always been a “fundamental cause” of mortality, but rather only emerged as a crucial determinant during the second half of the twentieth century, and especially after 1970. Moreover, when the gradient emerged, it did so for both genders and all causes of death. In addition, our findings raise some doubt that individuals with higher SES always have lower mortality; instead, they point towards a more nuanced picture in which the impact of SES depends on which coping mechanisms each SES group exploits to avoid risk factors at each point in time. Lifestyle factors and behavioral habits were most likely important mechanisms creating SES differences in mortality over the last two centuries.

## Electronic supplementary material

Below is the link to the electronic supplementary material.

Supplementary material 1 (DOCX 102 kb)
