# Supplementary material for: SES inequalities in cause-specific adult mortality: a study of the long-term trends using longitudinal individual data for Sweden (1813–2014)
Source: Eur J Epidemiol. 2020 Oct 1;35(11):1043–56. doi: 10.1007/s10654-020-00685-6 (PMC7695660; doi:10.1007/s10654-020-00685-6)
Supplement: Supplementary file 1 — Supplementary material 1 (DOCX 102 kb) [file 10654_2020_685_MOESM1_ESM.docx]

**Supplementary Table 1.**

Ill-defined and missing groups, men, ages 30-90 (five parishes and, from 1922, Landskrona).

|  | Ill-defined causes | | Missing |
| --- | --- | --- | --- |
|  | 1813-1921 | 1922-1967 | 1813-1921 |
| Nonmanual | 0.606^*^ | 1.247 | 0.632^*^ |
| Manual (ref.) | 1 | 1 | 1 |
| Farmer | 0.823 | 1.358 | 0.833 |
| NA | 0.954 | 1.118 | 0.903 |
|  |  |  |  |
| N of subjects | 8141 | 25354 | 8141 |
| N of failures (deaths) | 551 | 397 | 634 |
| Time at risk (person years) | 99016.7 | 344954.4 | 99016.7 |

^*^ *p* < 0.05, ^**^ *p* < 0.01, ^***^ *p* < 0.001

**Supplementary Table 2.**

Ill-defined and missing groups, women, ages 30-90 (five parishes and, from 1922, Landskrona).

|  | Ill-defined causes | | Missing |
| --- | --- | --- | --- |
|  | 1813-1921 | 1922-1967 | 1813-1921 |
| Nonmanual | 0.887 | 0.817 | 0.748 |
| Manual (ref.) | 1 | 1 | 1 |
| Farmer | 0.720^**^ | 1.086 | 0.909 |
| NA | 1.179 | 0.929 | 1.279 |
|  |  |  |  |
| N of subjects | 8131 | 24213 | 8131 |
| N of failures (deaths) | 615 | 503 | 729 |
| Time at risk (person years) | 104349.9 | 366401.3 | 104349.9 |

^*^ *p* < 0.05, ^**^ *p* < 0.01, ^***^ *p* < 0.001

**Analysis with the six-category SES classification**

**Supplementary table 3.**

Nonpreventable vs preventable mortality, men, 30-90, more detailed SES groups (five parishes and, from 1922, Landskrona).

|  | Nonpreventable causes | | | | Preventable causes | | | |
| --- | --- | --- | --- | --- | --- | --- | --- | --- |
|  | 1813-1921 | 1922-1967 | 1968-1989 | 1990-2014 | 1813-1921 | 1922-1967 | 1968-1989 | 1990-2014 |
| Higher managers/professionals | 1.616 | 1.023 | 0.567^***^ | 0.595^***^ | 1.242 | 1.083 | 0.624^***^ | 0.467^***^ |
| Lower managers/professionals/clerical | 0.909 | 1.086 | 0.740^***^ | 0.604^***^ | 1.471 | 1.176^**^ | 0.692^***^ | 0.538^***^ |
| Foremen and medium skilled workers | 1.000 | 0.921 | 0.903 | 0.912 | 1.081 | 0.961 | 0.936 | 0.819^***^ |
| Lower skilled workers/farm workers (ref.) | 1 | 1 | 1 | 1 | 1 | 1 | 1 | 1 |
| Unskilled workers/farm workers | 1.118 | 0.988 | 1.294^*^ | 1.471^***^ | 1.159 | 1.064 | 1.051 | 1.282^***^ |
| Farmers and fishermen | 1.145 | 0.963 | 0.862 | 0.656^**^ | 0.890 | 1.224^*^ | 0.702^***^ | 0.685^***^ |
| NA | 1.393 | 1.322 | 1.179 | 1.329^*^ | 1.550 | 0.930 | 0.705 | 0.919 |
|  |  |  |  |  |  |  |  |  |
| N of subjects | 8141 | 25354 | 24369 | 34266 | 8141 | 25354 | 24369 | 34266 |
| N of failures (deaths) | 463 | 1336 | 1227 | 2011 | 361 | 2856 | 3536 | 4024 |
| Time at risk (person years) | 99016.7 | 344954.4 | 274102.4 | 379639.0 | 99016.7 | 344954.4 | 274102.4 | 379639.0 |

^*^ *p* < 0.05, ^**^ *p* < 0.01, ^***^ *p* < 0.001

**Supplementary table 4.**

Nonpreventable vs preventable mortality, women, 30-90, more detailed SES groups (five parishes and, from 1922, Landskrona).

|  | Nonpreventable causes | | | | Preventable causes | | | |
| --- | --- | --- | --- | --- | --- | --- | --- | --- |
|  | 1813-1921 | 1922-1967 | 1968-1989 | 1990-2014 | 1813-1921 | 1922-1967 | 1968-1989 | 1990-2014 |
| Higher managers/professionals | 1.191 | 1.026 | 0.508^***^ | 0.526^***^ | 0.747 | 0.775^*^ | 0.610^***^ | 0.438^***^ |
| Lower managers/professionals/clerical | 0.730 | 0.910 | 0.670^***^ | 0.694^***^ | 0.967 | 0.949 | 0.665^***^ | 0.659^***^ |
| Foremen and medium skilled workers | 0.888 | 0.898 | 0.722^***^ | 0.809^*^ | 1.322 | 1.014 | 0.712^***^ | 0.844^**^ |
| Lower skilled workers/farm workers (ref.) | 1 | 1 | 1 | 1 | 1 | 1 | 1 | 1 |
| Unskilled workers/farm workers | 0.967 | 1.118 | 1.222^*^ | 1.295^***^ | 0.953 | 1.038 | 1.409^***^ | 1.306^***^ |
| Farmers and fishermen | 1.265 | 0.847 | 0.688 | 0.527^***^ | 1.019 | 1.089 | 0.575^***^ | 0.631^***^ |
| NA | 1.184 | 1.190 | 0.695 | 1.333^*^ | 1.318 | 1.094 | 0.907 | 1.093 |
|  |  |  |  |  |  |  |  |  |
| N of subjects | 8131 | 24213 | 23252 | 33018 | 8131 | 24213 | 23252 | 33018 |
| N of failures (deaths) | 432 | 1170 | 971 | 1856 | 353 | 2624 | 2720 | 3431 |
| Time at risk (person years) | 104349.9 | 366401.3 | 291272.2 | 399771.6 | 104349.9 | 366401.3 | 291272.2 | 399771.6 |

^*^ *p* < 0.05, ^**^ *p* < 0.01, ^***^ *p* < 0.001

**Supplementary table 5.**

Cause-specific mortality, men, 30-90, more detailed SES groups (five parishes and, from 1922, Landskrona).

|  | Infectious and parasitic | | | | Circulatory system | | | | Respiratory system and lung cancer | | | |
| --- | --- | --- | --- | --- | --- | --- | --- | --- | --- | --- | --- | --- |
|  | 1813-1921 | 1922-1967 | 1968-1989 | 1990-2014 | 1813-1921 | 1922-1967 | 1968-1989 | 1990-2014 | 1813-1921 | 1922-1967 | 1968-1989 | 1990-2014 |
| Higher managers/professionals | 1.039 | 0.942 | 0.426 | 0.560 | 3.422^**^ | 1.237^*^ | 0.687^***^ | 0.515^***^ | 1.699 | 0.623 | 0.339^***^ | 0.357^***^ |
| Lower managers/professionals/clerical | 0.966 | 1.077 | 0.896 | 0.533^***^ | 2.236^*^ | 1.204^**^ | 0.730^***^ | 0.546^***^ | 0.441 | 1.146 | 0.579^***^ | 0.488^***^ |
| Foremen and medium skilled workers | 1.176 | 0.920 | 1.228 | 0.964 | 1.285 | 0.976 | 0.909 | 0.805^***^ | 0.912 | 0.879 | 0.981 | 0.863 |
| Lower skilled workers/farm workers (ref.) | 1 | 1 | 1 | 1 | 1 | 1 | 1 | 1 | 1 | 1 | 1 | 1 |
| Unskilled workers/farm workers | 1.370 | 1.473^**^ | 1.559 | 1.366 | 0.899 | 0.925 | 1.036 | 1.284^**^ | 1.233 | 0.842 | 1.064 | 1.411^*^ |
| Farmers and fishermen | 1.200 | 1.409 | 0.854 | 0.958 | 0.643 | 0.995 | 0.728^**^ | 0.651^***^ | 0.843 | 1.072 | 0.584 | 0.334^***^ |
| NA | 1.373 | 2.032^*^ | 1.027 | 1.363 | 1.121 | 0.739 | 0.773 | 0.808 | 1.153 | 0.610 | 0.927 | 1.131 |
|  |  |  |  |  |  |  |  |  |  |  |  |  |
| N of subjects | 8141 | 25354 | 24369 | 34266 | 8141 | 25354 | 24369 | 34266 | 8141 | 25354 | 24369 | 34266 |
| N of failures (deaths) | 291 | 474 | 163 | 233 | 99 | 1775 | 2510 | 2609 | 136 | 199 | 390 | 719 |
| Time at risk (person years) | 99016.7 | 344954.4 | 274102.4 | 379639.0 | 99016.7 | 344954.4 | 274102.4 | 379639.0 | 99016.7 | 344954.4 | 274102.4 | 379639.0 |

**Supplementary table 5** – continued.

|  | Other cancers | | | | External causes | | | | Other and ill-defined causes | | | |
| --- | --- | --- | --- | --- | --- | --- | --- | --- | --- | --- | --- | --- |
|  | 1813-1921 | 1922-1967 | 1968-1989 | 1990-2014 | 1813-1921 | 1922-1967 | 1968-1989 | 1990-2014 | 1813-1921 | 1922-1967 | 1968-1989 | 1990-2014 |
| Higher managers/professionals |  | 1.401^*^ | 0.688^*^ | 0.665^***^ |  | 0.526^*^ | 0.343^***^ | 0.340^***^ | 0.930 | 1.018 | 0.580^*^ | 0.410^***^ |
| Lower managers/professionals/clerical |  | 1.309^*^ | 0.810^*^ | 0.610^***^ |  | 0.747 | 0.417^***^ | 0.590^***^ | 0.647 | 1.268^*^ | 0.703^**^ | 0.588^***^ |
| Foremen and medium skilled workers |  | 1.097 | 0.923 | 0.830^*^ |  | 0.808 | 0.801 | 1.105 | 0.735 | 1.005 | 1.017 | 0.896 |
| Lower skilled workers/farm workers (ref.) |  | 1 | 1 | 1 |  | 1 | 1 | 1 | 1 | 1 | 1 | 1 |
| Unskilled workers/farm workers |  | 1.290^*^ | 1.243 | 1.529^***^ |  | 0.995 | 1.188 | 1.497 | 0.991 | 0.996 | 1.273 | 1.341^*^ |
| Farmers and fishermen |  | 1.246 | 0.835 | 0.791 |  | 0.925 | 0.697 | 1.353 | 0.861 | 1.387^*^ | 0.735 | 0.618^*^ |
| NA |  | 0.774 | 0.692 | 1.242 |  | 0.405 | 0.828 | 1.321 | 1.161 | 1.543 | 1.163 | 1.109 |
|  |  |  |  |  |  |  |  |  |  |  |  |  |
| N of subjects |  | 25354 | 24369 | 34266 |  | 25354 | 24369 | 34266 | 8141 | 25354 | 24369 | 34266 |
| N of failures (deaths) |  | 792 | 909 | 1333 |  | 388 | 366 | 317 | 718 | 961 | 437 | 896 |
| Time at risk (person years) |  | 344954.4 | 274102.4 | 379639.0 |  | 344954.4 | 274102.4 | 379639.0 | 99016.7 | 344954.4 | 274102.4 | 379639.0 |

Exponentiated coefficients; ^*^ *p* < 0.05, ^**^ *p* < 0.01, ^***^ *p* < 0.001

**Supplementary table 6.**

Cause-specific mortality, women, 30-90, more detailed SES groups (five parishes and, from 1922, Landskrona).

|  | Infectious and parasitic | | | | Circulatory system | | | | Respiratory system and lung cancer | | | |
| --- | --- | --- | --- | --- | --- | --- | --- | --- | --- | --- | --- | --- |
|  | 1813-1921 | 1922-1967 | 1968-1989 | 1990-2014 | 1813-1921 | 1922-1967 | 1968-1989 | 1990-2014 | 1813-1921 | 1922-1967 | 1968-1989 | 1990-2014 |
| Higher managers/professionals | 0.892 | 1.039 | 0.889 | 0.520 | 1.656 | 0.811 | 0.608^***^ | 0.582^***^ |  | 0.397 | 0.509 | 0.231^***^ |
| Lower managers/professionals/clerical | 1.199 | 0.726^*^ | 0.570^*^ | 0.735 | 1.258 | 1.013 | 0.672^***^ | 0.682^***^ |  | 0.754 | 0.585^**^ | 0.626^***^ |
| Foremen and medium skilled workers | 0.967 | 1.043 | 0.736 | 0.779 | 1.500 | 1.029 | 0.784^***^ | 0.869 |  | 0.802 | 0.687 | 0.761 |
| Farmers and fishermen | 1.200 | 1.202 | 0.577 | 0.256 | 1.038 | 1.293^*^ | 0.791 | 0.663^**^ |  | 0.394 | 0.146 | 0.564 |
| Lower skilled workers/farm workers (ref.) | 1 | 1 | 1 | 1 | 1 | 1 | 1 | 1 |  | 1 | 1 | 1 |
| Unskilled workers/farm workers | 1.058 | 1.247 | 1.534 | 1.041 | 0.755 | 1.080 | 1.286^***^ | 1.166^*^ |  | 0.500^*^ | 1.057 | 1.541^***^ |
| Farmers and fishermen | 1.200 | 1.202 | 0.577 | 0.256 | 1.038 | 1.293^*^ | 0.791 | 0.663^**^ |  | 0.394 | 0.146 | 0.564 |
| NA | 1.273 | 0.891 | 0.745 | 1.694 | 1.889 | 1.085 | 0.897 | 1.278^*^ |  | 1.033 | 0.436 | 0.609 |
|  |  |  |  |  |  |  |  |  |  |  |  |  |
| N of subjects | 8131 | 24213 | 23252 | 33018 | 8131 | 24213 | 23252 | 33018 |  | 24213 | 23252 | 33018 |
| N of failures (deaths) | 297 | 451 | 161 | 241 | 101 | 1692 | 1844 | 2193 |  | 136 | 180 | 508 |
| Time at risk (person years) | 104349.9 | 366401.3 | 291272.2 | 399771.6 | 104349.9 | 366401.3 | 291272.2 | 399771.6 |  | 366401.3 | 291272.2 | 399771.6 |

**Supplementary table 6** – continued.

|  | Other cancers | | | | External causes | | | | Other and ill-defined causes | | | |
| --- | --- | --- | --- | --- | --- | --- | --- | --- | --- | --- | --- | --- |
|  | 1813-1921 | 1922-1967 | 1968-1989 | 1990-2014 | 1813-1921 | 1922-1967 | 1968-1989 | 1990-2014 | 1813-1921 | 1922-1967 | 1968-1989 | 1990-2014 |
| Higher managers/professionals | 1.358 | 0.806 | 0.474^***^ | 0.376^***^ |  | 1.294 | 0.933 | 0.611 | 1.036 | 0.867 | 0.532^*^ | 0.530^**^ |
| Lower managers/professionals/clerical | 1.013 | 0.975 | 0.685^***^ | 0.655^***^ |  | 0.888 | 0.619^*^ | 0.620^*^ | 0.634 | 0.875 | 0.673^**^ | 0.695^***^ |
| Foremen and medium skilled workers | 1.792 | 1.007 | 0.604^***^ | 0.640^***^ |  | 1.102 | 0.670 | 1.199 | 0.798 | 0.842 | 0.683^*^ | 1.084 |
| Lower skilled workers/farm workers (ref.) | 1 | 1 | 1 | 1 |  | 1 | 1 | 1 | 1 | 1 | 1 | 1 |
| Unskilled workers/farm workers | 1.344 | 0.932 | 1.586^***^ | 1.358^***^ |  | 1.327 | 1.692^*^ | 1.788^**^ | 0.881 | 1.160 | 1.289 | 1.511^***^ |
| Farmers and fishermen | 1.014 | 0.702 | 0.387^***^ | 0.577^*^ |  | 0.480 | 0.605 | 1.061 | 0.807^*^ | 0.919 | 0.587 | 0.532^*^ |
| NA | 2.177 | 1.270 | 0.769 | 1.022 |  | 1.974 | 1.407 | 0.737 | 0.998 | 1.038 | 0.801 | 1.521^**^ |
|  |  |  |  |  |  |  |  |  |  |  |  |  |
| N of subjects | 8131 | 24213 | 23252 | 33018 |  | 24213 | 23252 | 33018 | 8131 | 24213 | 23252 | 33018 |
| N of failures (deaths) | 90 | 862 | 920 | 1267 |  | 132 | 168 | 181 | 784 | 1024 | 423 | 930 |
| Time at risk (person years) | 104349.9 | 366401.3 | 291272.2 | 399771.6 |  | 366401.3 | 291272.2 | 399771.6 | 104349.9 | 366401.3 | 291272.2 | 399771.6 |

Exponentiated coefficients; ^*^ *p* < 0.05, ^**^ *p* < 0.01, ^***^ *p* < 0.001

**Sensitivity analyses**

Table A1: Nonpreventable vs preventable mortality, men, 30-90, only five parishes

|  | Nonpreventable causes | | | | Preventable causes | | | |
| --- | --- | --- | --- | --- | --- | --- | --- | --- |
|  | 1813-1921 | 1922-1967 | 1968-1989 | 1990-2014 | 1813-1921 | 1922-1967 | 1968-1989 | 1990-2014 |
| Nonmanual | 1.117 | 1.052 | 0.736 | 0.682^***^ | 1.299 | 1.181 | 0.775^**^ | 0.563^***^ |
| Manual (ref.) | 1 | 1 | 1 | 1 | 1 | 1 | 1 | 1 |
| Farmer | 1.099 | 0.853 | 0.938 | 0.644^*^ | 0.827 | 1.059 | 0.821 | 0.772^*^ |
| NA | 1.324 | 1.180 | 0.591 | 2.648^**^ | 1.427 | 1.157 | 0.839 | 0.856 |
|  |  |  |  |  |  |  |  |  |
| N of subjects | 8141 | 6886 | 5125 | 8381 | 8141 | 6886 | 5125 | 8381 |
| N of failures (deaths) | 463 | 326 | 206 | 417 | 361 | 644 | 625 | 788 |
| Time at risk (person years) | 99016.7 | 82440.6 | 48804.4 | 79264.0 | 99016.7 | 82440.6 | 48804.4 | 79264.0 |

^*^ *p* < 0.05, ^**^ *p* < 0.01, ^***^ *p* < 0.001

Table A2: Nonpreventable vs preventable mortality, women, 30-90, only five parishes

|  | Nonpreventable causes | | | | Preventable causes | | | |
| --- | --- | --- | --- | --- | --- | --- | --- | --- |
|  | 1813-1921 | 1922-1967 | 1968-1989 | 1990-2014 | 1813-1921 | 1922-1967 | 1968-1989 | 1990-2014 |
| Nonmanual | 0.919 | 0.886 | 0.514^**^ | 0.761^*^ | 0.853 | 0.925 | 0.533^***^ | 0.683^***^ |
| Manual (ref.) | 1 | 1 | 1 | 1 | 1 | 1 | 1 | 1 |
| Farmer | 1.298^*^ | 0.787 | 0.841 | 0.745 | 0.988 | 1.058 | 0.613^**^ | 0.529^***^ |
| NA | 1.209 | 1.481 | 0.891 | 1.924^*^ | 1.268 | 0.843 | 0.720 | 1.252 |
|  |  |  |  |  |  |  |  |  |
| N of subjects | 8131 | 6410 | 4778 | 8181 | 8131 | 6410 | 4778 | 8181 |
| N of failures (deaths) | 432 | 309 | 174 | 375 | 353 | 617 | 448 | 677 |
| Time at risk (person years) | 104349.9 | 81511.0 | 49293.4 | 82624.6 | 104349.9 | 81511.0 | 49293.4 | 82624.6 |

^*^ *p* < 0.05, ^**^ *p* < 0.01, ^***^ *p* < 0.001

Table A3: Nonpreventable vs preventable mortality, men, 30-90, entire sample

|  | Nonpreventable causes | | | | Preventable causes | | | |
| --- | --- | --- | --- | --- | --- | --- | --- | --- |
|  | 1813-1921 | 1922-1967 | 1968-1989 | 1990-2014 | 1813-1921 | 1922-1967 | 1968-1989 | 1990-2014 |
| Nonmanual | 1.117 | 1.106 | 0.684^***^ | 0.666^***^ | 1.299 | 1.150^**^ | 0.701^***^ | 0.620^***^ |
| Manual (ref.) | 1 | 1 | 1 | 1 | 1 | 1 | 1 | 1 |
| Farmer | 1.099 | 0.990 | 0.617^***^ | 0.745^***^ | 0.827 | 1.209^*^ | 0.650^***^ | 0.695^***^ |
| NA | 1.324 | 1.361 | 1.431^***^ | 1.829^***^ | 1.427 | 0.920 | 1.261^***^ | 1.318^***^ |
|  |  |  |  |  |  |  |  |  |
| N of subjects | 8141 | 25354 | 145869 | 219824 | 8141 | 25354 | 145869 | 219824 |
| N of failures (deaths) | 463 | 1336 | 4918 | 15776 | 361 | 2856 | 14370 | 29492 |
| Time at risk (person years) | 99016.7 | 344954.4 | 2193893.9 | 3652338.1 | 99016.7 | 344954.4 | 2193893.9 | 3652338.1 |

^*^ *p* < 0.05, ^**^ *p* < 0.01, ^***^ *p* < 0.001

Table A4: Nonpreventable vs preventable mortality, women, 30-90, entire sample

|  | Nonpreventable causes | | | | Preventable causes | | | |
| --- | --- | --- | --- | --- | --- | --- | --- | --- |
|  | 1813-1921 | 1922-1967 | 1968-1989 | 1990-2014 | 1813-1921 | 1922-1967 | 1968-1989 | 1990-2014 |
| Nonmanual | 0.919 | 0.944 | 0.657^***^ | 0.691^***^ | 0.853 | 0.901^*^ | 0.598^***^ | 0.629^***^ |
| Manual (ref.) | 1 | 1 | 1 | 1 | 1 | 1 | 1 | 1 |
| Farmer | 1.298^*^ | 0.847 | 0.602^***^ | 0.659^***^ | 0.988 | 1.075 | 0.515^***^ | 0.630^***^ |
| NA | 1.209 | 1.199 | 1.113 | 1.227^***^ | 1.268 | 1.077 | 1.111^**^ | 1.200^***^ |
|  |  |  |  |  |  |  |  |  |
| N of subjects | 8131 | 24213 | 146493 | 224037 | 8131 | 24213 | 146493 | 224037 |
| N of failures (deaths) | 432 | 1170 | 3440 | 14219 | 353 | 2624 | 8710 | 25298 |
| Time at risk (person years) | 104349.9 | 366401.3 | 2287749.2 | 3868485.8 | 104349.9 | 366401.3 | 2287749.2 | 3868485.8 |

^*^ *p* < 0.05, ^**^ *p* < 0.01, ^***^ *p* < 0.001

Table A5: Nonpreventable vs preventable mortality, men, 30-90, five parishes and Landskrona, excluding those not born in Sweden

|  | Nonpreventable causes | | | | Preventable causes | | | |
| --- | --- | --- | --- | --- | --- | --- | --- | --- |
|  | 1813-1921 | 1922-1967 | 1968-1989 | 1990-2014 | 1813-1921 | 1922-1967 | 1968-1989 | 1990-2014 |
| Nonmanual | 1.138 | 1.112 | 0.704^***^ | 0.605^***^ | 1.347 | 1.143^**^ | 0.703^***^ | 0.568^***^ |
| Manual (ref.) | 1 | 1 | 1 | 1 | 1 | 1 | 1 | 1 |
| Farmer | 1.089 | 0.993 | 0.883 | 0.636^**^ | 0.824 | 1.194 | 0.704^***^ | 0.734^***^ |
| NA | 1.321 | 1.181 | 1.511 | 2.706^***^ | 1.424 | 0.932 | 1.168 | 0.968 |
|  |  |  |  |  |  |  |  |  |
| N of subjects | 7967 | 22998 | 20522 | 26067 | 7967 | 22998 | 20522 | 26067 |
| N of failures (deaths) | 462 | 1286 | 1141 | 1723 | 357 | 2786 | 3338 | 3474 |
| Time at risk (person years) | 97864.9 | 329613.0 | 239671.7 | 308717.1 | 97864.9 | 329613.0 | 239671.7 | 308717.1 |

^*^ *p* < 0.05, ^**^ *p* < 0.01, ^***^ *p* < 0.001

Table A6: Nonpreventable vs preventable mortality, women, 30-90, five parishes and Landskrona, excluding those not born in Sweden

|  | Nonpreventable causes | | | | Preventable causes | | | |
| --- | --- | --- | --- | --- | --- | --- | --- | --- |
|  | 1813-1921 | 1922-1967 | 1968-1989 | 1990-2014 | 1813-1921 | 1922-1967 | 1968-1989 | 1990-2014 |
| Nonmanual | 0.930 | 0.945 | 0.696^***^ | 0.684^***^ | 0.870 | 0.899^*^ | 0.669^***^ | 0.613^***^ |
| Manual (ref.) | 1 | 1 | 1 | 1 | 1 | 1 | 1 | 1 |
| Farmer | 1.300^*^ | 0.845 | 0.753 | 0.528^***^ | 0.979 | 1.063 | 0.583^***^ | 0.611^***^ |
| NA | 1.218 | 1.210 | 0.801 | 1.474^*^ | 1.286 | 1.104 | 1.069 | 1.215 |
|  |  |  |  |  |  |  |  |  |
| N of subjects | 7987 | 22383 | 20095 | 25795 | 7987 | 22383 | 20095 | 25795 |
| N of failures (deaths) | 432 | 1142 | 905 | 1606 | 351 | 2554 | 2565 | 2977 |
| Time at risk (person years) | 103454.0 | 351456.1 | 258221.9 | 325369.2 | 103454.0 | 351456.1 | 258221.9 | 325369.2 |

^*^ *p* < 0.05, ^**^ *p* < 0.01, ^***^ *p* < 0.001

Table A7: Nonpreventable vs preventable mortality, men, 30-90, five parishes and Landskrona, individual SES

|  | Nonpreventable causes | | | | Preventable causes | | | |
| --- | --- | --- | --- | --- | --- | --- | --- | --- |
|  | 1813-1921 | 1922-1967 | 1968-1989 | 1990-2014 | 1813-1921 | 1922-1967 | 1968-1989 | 1990-2014 |
| Nonmanual | 1.064 | 1.040 | 0.915 | 0.875^**^ | 1.317 | 1.090^*^ | 0.903^**^ | 0.806^***^ |
| Manual (ref.) | 1 | 1 | 1 | 1 | 1 | 1 | 1 | 1 |
| Farmer | 1.150 | 0.979 | 0.906 | 0.708^*^ | 0.821 | 1.116 | 0.758^**^ | 0.781^**^ |
| NA | 1.448 | 1.367 | 1.357 | 1.520^***^ | 1.451 | 0.982 | 0.837 | 1.077 |
|  |  |  |  |  |  |  |  |  |
| N of subjects | 8141 | 25354 | 24369 | 34266 | 8141 | 25354 | 24369 | 34266 |
| N of failures (deaths) | 463 | 1336 | 1227 | 2011 | 361 | 2856 | 3536 | 4024 |
| Time at risk (person years) | 99016.7 | 344954.4 | 274102.4 | 379639.0 | 99016.7 | 344954.4 | 274102.4 | 379639.0 |

^*^ *p* < 0.05, ^**^ *p* < 0.01, ^***^ *p* < 0.001

Table A8: Nonpreventable vs preventable mortality, women, 30-90, five parishes and Landskrona, individual SES

|  | Nonpreventable causes | | | | Preventable causes | | | |
| --- | --- | --- | --- | --- | --- | --- | --- | --- |
|  | 1813-1921 | 1922-1967 | 1968-1989 | 1990-2014 | 1813-1921 | 1922-1967 | 1968-1989 | 1990-2014 |
| Nonmanual | 0.912 | 0.936 | 0.897 | 0.877^*^ | 0.762 | 0.921 | 0.853^***^ | 0.778^***^ |
| Manual (ref.) | 1 | 1 | 1 | 1 | 1 | 1 | 1 | 1 |
| Farmer | 1.261 | 0.921 | 1.074 | 0.649^*^ | 1.016 | 1.104 | 0.838 | 0.817 |
| NA | 1.380 | 1.180 | 1.055 | 1.301^**^ | 1.228 | 1.077 | 1.025 | 1.115 |
|  |  |  |  |  |  |  |  |  |
| N of subjects | 8131 | 24213 | 23252 | 33018 | 8131 | 24213 | 23252 | 33018 |
| N of failures (deaths) | 432 | 1170 | 971 | 1856 | 353 | 2624 | 2720 | 3431 |
| Time at risk (person years) | 104349.9 | 366401.3 | 291272.2 | 399771.6 | 104349.9 | 366401.3 | 291272.2 | 399771.6 |

^*^ *p* < 0.05, ^**^ *p* < 0.01, ^***^ *p* < 0.001

Table A9: Nonpreventable vs preventable mortality, men, 30-69, five parishes and Landskrona

|  | Nonpreventable causes | | | | Preventable causes | | | |
| --- | --- | --- | --- | --- | --- | --- | --- | --- |
|  | 1813-1921 | 1922-1967 | 1968-1989 | 1990-2014 | 1813-1921 | 1922-1967 | 1968-1989 | 1990-2014 |
| Nonmanual | 1.019 | 1.018 | 0.670^***^ | 0.677^***^ | 1.292 | 1.146^*^ | 0.599^***^ | 0.440^***^ |
| Manual (ref.) | 1 | 1 | 1 | 1 | 1 | 1 | 1 | 1 |
| Farmer | 1.125 | 1.124 | 0.856 | 0.543 | 0.742 | 1.052 | 0.693^*^ | 0.843 |
| NA | 1.297 | 1.639 | 1.168 | 1.783^***^ | 1.573 | 1.299 | 0.606^*^ | 0.876 |
|  |  |  |  |  |  |  |  |  |
| N of subjects | 8141 | 25219 | 23114 | 31210 | 8141 | 25219 | 23114 | 31210 |
| N of failures (deaths) | 413 | 903 | 595 | 631 | 300 | 1654 | 1536 | 1213 |
| Time at risk (person years) | 91937.2 | 318596.9 | 237797.3 | 312809.9 | 91937.2 | 318596.9 | 237797.3 | 312809.9 |

^*^ *p* < 0.05, ^**^ *p* < 0.01, ^***^ *p* < 0.001

Table A10: Nonpreventable vs preventable mortality, men, 70-90, five parishes and Landskrona

|  | Nonpreventable causes | | | | Preventable causes | | | |
| --- | --- | --- | --- | --- | --- | --- | --- | --- |
|  | 1813-1921 | 1922-1967 | 1968-1989 | 1990-2014 | 1813-1921 | 1922-1967 | 1968-1989 | 1990-2014 |
| Nonmanual | 1.896 | 1.352^**^ | 0.773^**^ | 0.573^***^ | 1.420 | 1.157^*^ | 0.797^***^ | 0.614^***^ |
| Manual (ref.) | 1 | 1 | 1 | 1 | 1 | 1 | 1 | 1 |
| Farmer | 0.814 | 0.806 | 0.842 | 0.660^**^ | 1.183 | 1.282^*^ | 0.707^**^ | 0.714^***^ |
| NA | 1.482 | 1.058 | 1.190 | 0.955 | 0.483 | 0.644 | 0.909 | 1.056 |
|  |  |  |  |  |  |  |  |  |
| N of subjects | 898 | 3367 | 5356 | 8806 | 898 | 3367 | 5356 | 8806 |
| N of failures (deaths) | 50 | 433 | 632 | 1380 | 61 | 1202 | 2000 | 2811 |
| Time at risk (person years) | 7079.6 | 26357.5 | 36305.1 | 66829.2 | 7079.6 | 26357.5 | 36305.1 | 66829.2 |

^*^ *p* < 0.05, ^**^ *p* < 0.01, ^***^ *p* < 0.001

Table A11: Nonpreventable vs preventable mortality, women, 30-69, five parishes and Landskrona

|  | Nonpreventable causes | | | | Preventable causes | | | |
| --- | --- | --- | --- | --- | --- | --- | --- | --- |
|  | 1813-1921 | 1922-1967 | 1968-1989 | 1990-2014 | 1813-1921 | 1922-1967 | 1968-1989 | 1990-2014 |
| Nonmanual | 0.881 | 0.886 | 0.535^***^ | 0.457^***^ | 0.931 | 0.906 | 0.507^***^ | 0.525^***^ |
| Manual (ref.) | 1 | 1 | 1 | 1 | 1 | 1 | 1 | 1 |
| Farmer | 1.265 | 0.708 | 0.320^*^ | 0.512 | 1.090 | 0.943 | 0.301^***^ | 0.535 |
| NA | 1.151 | 1.152 | 0.493^*^ | 1.190 | 1.365 | 1.087 | 0.682 | 1.143 |
|  |  |  |  |  |  |  |  |  |
| N of subjects | 8131 | 24059 | 21566 | 28617 | 8131 | 24059 | 21566 | 28617 |
| N of failures (deaths) | 383 | 720 | 368 | 371 | 295 | 1330 | 778 | 736 |
| Time at risk (person years) | 96980.6 | 332516.8 | 238450.8 | 305430.7 | 96980.6 | 332516.8 | 238450.8 | 305430.7 |

^*^ *p* < 0.05, ^**^ *p* < 0.01, ^***^ *p* < 0.001

Table A12: Nonpreventable vs preventable mortality, women, 70-90, five parishes and Landskrona

|  | Nonpreventable causes | | | | Preventable causes | | | |
| --- | --- | --- | --- | --- | --- | --- | --- | --- |
|  | 1813-1921 | 1922-1967 | 1968-1989 | 1990-2014 | 1813-1921 | 1922-1967 | 1968-1989 | 1990-2014 |
| Nonmanual | 1.327 | 1.064 | 0.815^*^ | 0.733^***^ | 0.575 | 0.900 | 0.765^***^ | 0.649^***^ |
| Manual (ref.) | 1 | 1 | 1 | 1 | 1 | 1 | 1 | 1 |
| Farmer | 1.569 | 0.986 | 1.021 | 0.523^**^ | 0.482 | 1.164 | 0.722^*^ | 0.627^***^ |
| NA | 1.931 | 1.331 | 0.902 | 1.365^*^ | 0.759 | 1.076 | 1.014 | 1.030 |
|  |  |  |  |  |  |  |  |  |
| N of subjects | 993 | 4045 | 6793 | 10819 | 993 | 4045 | 6793 | 10819 |
| N of failures (deaths) | 49 | 450 | 603 | 1485 | 58 | 1294 | 1942 | 2695 |
| Time at risk (person years) | 7369.3 | 33884.5 | 52821.4 | 94340.9 | 7369.3 | 33884.5 | 52821.4 | 94340.9 |

^*^ *p* < 0.05, ^**^ *p* < 0.01, ^***^ *p* < 0.001

Table A13: Men, 30-90, five parishes and Landskrona, preventability according to Phelan et al. 2004

|  | Nonpreventable causes | | | | Preventable causes | | | |
| --- | --- | --- | --- | --- | --- | --- | --- | --- |
|  | 1813-1921 | 1922-1967 | 1968-1989 | 1990-2014 | 1813-1921 | 1922-1967 | 1968-1989 | 1990-2014 |
| Nonmanual | 0.708 | 1.129 | 0.723^***^ | 0.645^***^ | 1.231 | 1.169^**^ | 0.704^***^ | 0.535^***^ |
| Manual (ref.) | 1 | 1 | 1 | 1 | 1 | 1 | 1 | 1 |
| Farmer | 0.860 | 1.101 | 0.781 | 0.716^*^ | 0.951 | 1.129 | 0.686^***^ | 0.646^***^ |
| NA | 1.210 | 0.647 | 0.831 | 0.954 | 1.470 | 1.120 | 0.825 | 0.962 |
|  |  |  |  |  |  |  |  |  |
| N of subjects | 8141 | 25354 | 24369 | 34266 | 8141 | 25354 | 24369 | 34266 |
| N of failures (deaths) | 109 | 822 | 839 | 1266 | 330 | 2088 | 2833 | 3401 |
| Time at risk (person years) | 99016.7 | 344954.4 | 274102.4 | 379639.0 | 99016.7 | 344954.4 | 274102.4 | 379639.0 |

^*^ *p* < 0.05, ^**^ *p* < 0.01, ^***^ *p* < 0.001

Table A14: Women, 30-90, five parishes and Landskrona, preventability according to Phelan et al. 2004

|  | Nonpreventable causes | | | | Preventable causes | | | |
| --- | --- | --- | --- | --- | --- | --- | --- | --- |
|  | 1813-1921 | 1922-1967 | 1968-1989 | 1990-2014 | 1813-1921 | 1922-1967 | 1968-1989 | 1990-2014 |
| Nonmanual | 0.850 | 0.973 | 0.668^***^ | 0.651^***^ | 0.952 | 0.942 | 0.677^***^ | 0.630^***^ |
| Manual (ref.) | 1 | 1 | 1 | 1 | 1 | 1 | 1 | 1 |
| Farmer | 0.931 | 0.850 | 0.608^*^ | 0.527^**^ | 1.126 | 1.176 | 0.590^**^ | 0.543^***^ |
| NA | 1.168 | 1.233 | 0.806 | 1.040 | 1.310 | 0.987 | 0.885 | 1.062 |
|  |  |  |  |  |  |  |  |  |
| N of subjects | 8131 | 24213 | 23252 | 33018 | 8131 | 24213 | 23252 | 33018 |
| N of failures (deaths) | 153 | 734 | 843 | 1230 | 320 | 1984 | 2050 | 2838 |
| Time at risk (person years) | 104349.9 | 366401.3 | 291272.2 | 399771.6 | 104349.9 | 366401.3 | 291272.2 | 399771.6 |

^*^ *p* < 0.05, ^**^ *p* < 0.01, ^***^ *p* < 0.001

Table A15: Men, 30-90, five parishes and Landskrona, SES measured with HISCAM quartiles calculated by sex and period

|  | Nonpreventable causes | | | | Preventable causes | | | |
| --- | --- | --- | --- | --- | --- | --- | --- | --- |
|  | 1813-1921 | 1922-1967 | 1968-1989 | 1990-2014 | 1813-1921 | 1922-1967 | 1968-1989 | 1990-2014 |
| Lowest quartile | 1.062 | 1.048 | 1.254^**^ | 1.258^***^ | 1.378 | 1.097 | 1.234^***^ | 1.272^***^ |
| Second quartile (ref.) | 1 | 1 | 1 | 1 | 1 | 1 | 1 | 1 |
| Third quartile | 1.031 | 0.988 | 0.944 | 0.891 | 1.331 | 1.046 | 0.881^*^ | 0.823^***^ |
| Highest quartile | 1.138 | 1.164 | 0.800^*^ | 0.668^***^ | 1.631^*^ | 1.158^**^ | 0.752^***^ | 0.553^***^ |
| NA | 1.311 | 1.292 | 1.168 | 1.303^*^ | 1.573 | 1.759^***^ | 0.800 | 1.093 |
|  |  |  |  |  |  |  |  |  |
| N of subjects | 8141 | 25354 | 24369 | 34266 | 8141 | 25354 | 24369 | 34266 |
| N of failures (deaths) | 463 | 1336 | 1227 | 2011 | 361 | 2856 | 3536 | 4024 |
| Time at risk (person years) | 99016.7 | 344954.4 | 274102.4 | 379639.0 | 99016.7 | 344954.4 | 274102.4 | 379639.0 |

^*^ *p* < 0.05, ^**^ *p* < 0.01, ^***^ *p* < 0.001

Table A16: Women, 30-90, five parishes and Landskrona, SES measured with HISCAM quartiles calculated by sex and period

|  | Nonpreventable causes | | | | Preventable causes | | | |
| --- | --- | --- | --- | --- | --- | --- | --- | --- |
|  | 1813-1921 | 1922-1967 | 1968-1989 | 1990-2014 | 1813-1921 | 1922-1967 | 1968-1989 | 1990-2014 |
| Lowest quartile | 0.854 | 1.013 | 1.224^*^ | 1.249^***^ | 1.292 | 0.980 | 1.337^***^ | 1.275^***^ |
| Second quartile (ref.) | 1 | 1 | 1 | 1 | 1 | 1 | 1 | 1 |
| Third quartile | 1.267 | 0.926 | 0.779^*^ | 0.859^*^ | 1.180 | 0.963 | 0.836^**^ | 0.874^*^ |
| Highest quartile | 0.957 | 0.898 | 0.746^*^ | 0.546^***^ | 1.088 | 0.928 | 0.648^***^ | 0.584^***^ |
| NA | 1.112 | 1.106 | 0.854 | 1.458^**^ | 1.359 | 1.167 | 1.034 | 1.227^*^ |
|  |  |  |  |  |  |  |  |  |
| N of subjects | 8131 | 24213 | 23252 | 33018 | 8131 | 24213 | 23252 | 33018 |
| N of failures (deaths) | 432 | 1170 | 971 | 1856 | 353 | 2624 | 2720 | 3431 |
| Time at risk (person years) | 104349.9 | 366401.3 | 291272.2 | 399771.6 | 104349.9 | 366401.3 | 291272.2 | 399771.6 |

^*^ *p* < 0.05, ^**^ *p* < 0.01, ^***^ *p* < 0.001

Table A17: Men, 30-90, five parishes and Landskrona, SES measured with HISCAM as continuous variable

|  | Nonpreventable causes | | | | Preventable causes | | | |
| --- | --- | --- | --- | --- | --- | --- | --- | --- |
|  | 1813-1921 | 1922-1967 | 1968-1989 | 1990-2014 | 1813-1921 | 1922-1967 | 1968-1989 | 1990-2014 |
| HISCAM score | 1.002 | 1.004 | 0.986^***^ | 0.984^***^ | 1.009 | 1.001 | 0.984^***^ | 0.976^***^ |
| N of subjects | 7708 | 24960 | 22793 | 29435 | 7708 | 24960 | 22793 | 29435 |
| N of failures (deaths) | 421 | 1320 | 1199 | 1906 | 337 | 2819 | 3491 | 3855 |
| Time at risk (person years) | 93192.6 | 340979.1 | 262664.0 | 346357.9 | 93192.6 | 340979.1 | 262664.0 | 346357.9 |

^*^ *p* < 0.05, ^**^ *p* < 0.01, ^***^ *p* < 0.001

Table A18: Women, 30-90, five parishes and Landskrona, SES measured with HISCAM as continuous variable

|  | Nonpreventable causes | | | | Preventable causes | | | |
| --- | --- | --- | --- | --- | --- | --- | --- | --- |
|  | 1813-1921 | 1922-1967 | 1968-1989 | 1990-2014 | 1813-1921 | 1922-1967 | 1968-1989 | 1990-2014 |
| HISCAM score | 1.010 | 0.997 | 0.981^***^ | 0.979^***^ | 0.997 | 0.997 | 0.978^***^ | 0.978^***^ |
| N of subjects | 7477 | 23134 | 21960 | 29528 | 7477 | 23134 | 21960 | 29528 |
| N of failures (deaths) | 385 | 1084 | 942 | 1738 | 323 | 2410 | 2634 | 3242 |
| Time at risk (person years) | 96273.4 | 350898.0 | 279277.6 | 370474.8 | 96273.4 | 350898.0 | 279277.6 | 370474.8 |

^*^ *p* < 0.05, ^**^ *p* < 0.01, ^***^ *p* < 0.001
